# Intermittent Pool Beds Are Permanent Cyclic Habitats with Distinct Wet, Moist and Dry Phases

**DOI:** 10.1371/journal.pone.0108203

**Published:** 2014-09-22

**Authors:** Anthony I. Dell, Ross A. Alford, Richard G. Pearson

**Affiliations:** 1 School of Marine and Tropical Biology, James Cook University, Townsville, Queensland, Australia; 2 Systemic Conservation Biology, Department of Biology, Georg-August University Göttingen, Göttingen, Germany; University of Waikato (National Institute of Water and Atmospheric Research), New Zealand

## Abstract

Recognition that intermittent pools are a single habitat phase of an intermittent pool bed that cycles between aquatic and terrestrial habitat greatly enhances their usefulness for addressing general questions in ecology. The aquatic phase has served as a model system in many ecological studies, because it has distinct habitat boundaries in space and time and is an excellent experimental system, but the aquatic to terrestrial transition and terrestrial phase remain largely unstudied. We conducted a field experiment within six replicate natural intermittent pool beds to explore macroinvertebrate community dynamics during the transition from aquatic to terrestrial habitat and during the terrestrial phase. We monitored and compared macroinvertebrate communities within leaf packs that i) remained wet, ii) underwent drying (i.e., started wet and then dried), and iii) remained dry. Our results show that i) a diverse macroinvertebrate community inhabits all phases of intermittent pool beds, ii) pool drying involves colonization by an assemblage of macroinvertebrates not recorded in permanently terrestrial leaf packs, iii) the community within dried leaf packs remains distinct from that of permanently terrestrial leaf packs for an extended period following drying (possibly until subsequent refilling), and iv) there are likely to be strong spatial and temporal resource linkages between the aquatic and terrestrial communities. The unique environmental characteristics of intermittent pool beds, which repeatedly cycle from aquatic to terrestrial habitat, should continue to make them valuable study systems.

## Introduction

Intermittent pools are natural bodies of water that periodically dry, and often occur within intermittent streambeds following cessation of surface flow. Generally they are viewed as small, isolated patches of temporary aquatic habitat that undergo repetitive episodes of colonization and extinction. Because intermittent pools are bordered by distinct aquatic-terrestrial habitat boundaries in both space (at their aquatic margins) and time (during pool drying and filling), and because they are excellent experimental systems [1], they are often used as model systems for ecological studies. Insights gained from research on intermittent pools have enhanced our understanding of predation [2]–[5], competition [6], [7], trophic functionality [8]–[10], metapopulation dynamics [11]–[13], trophic cascades [14], [15] and food web dynamics [16]–[20]. However, the perspective that intermittent pools are temporally and spatially isolated temporary aquatic habitats constrains the range of ecological questions that can be addressed by studying them.

An alternative view is that the substrate over which intermittent pools form is a permanent fixture within the terrestrial landscape – one that experiences recurrent shifts from aquatic to terrestrial habitat [21], [22]. Thus, intermittent pools are a single habitat phase of a larger and more complex permanent cyclic habitat: an intermittent pool bed. Intermittent pool beds consist of both intermittent aquatic systems that periodically dry, and intermittent terrestrial systems that are periodically inundated. The existence of the intermittent terrestrial phase is implied by the existence of the intermittent aquatic phase, but the majority of existing ecological studies on intermittent pool beds have begun with the filling of the pool and ceased with its drying [23], [24]. Recognition that intermittent pools are a single phase of an environment that cycles between aquatic and terrestrial phases greatly increases their usefulness as a model study system for investigating general ecological principles [25].

One key area where study of intermittent pool beds should be productive is in understanding how the physical environment structures ecological systems. Traditional approaches to such questions involve comparisons of datasets collected from diverse habitats around the globe [26]–[31]. However, such comparisons are subject to confounding sources of variation that may mask or alter real patterns because datasets generally differ in how they were collated and constructed [32]–[35]. Because intermittent pool beds periodically and predictably cycle between aquatic and terrestrial phases in the same location, they provide a unique opportunity to examine ecological differences between very different habitats in a way that should be immune to many potentially confounding sources of variation. Thus, intermittent pool beds should be useful for addressing basic questions about ecological differences between aquatic and terrestrial habitats, such as patterns of colonization and extinction, food web architecture, and the role of habitat duration. They could also help understand the mechanics of energy and information transfer across habitat boundaries [36]–[42]. In this context intermittent pool beds are novel because not only are organisms and nutrients exchanged spatially between aquatic and terrestrial habits at their margins [25], but also temporally as the pool bed fills or dries [43]–[47]. Study of the transfer of resources across this ‘temporal ecotone’ should complement research on the spatial transfer of resources across habitat boundaries [22], [24], [48].

Although much is known about the aquatic phase of intermittent pool beds, the phase following surface water loss is poorly understood. For some time the pool bed substrate remains wetter than the surrounding terrestrial substrate, and during this time there can be important interactions between remnant aquatic and invading terrestrial species [21], [46], [49]–[54]. However, our understanding of the ecological dynamics during this transition is limited, and is based primarily on qualitative data [43]–[47], [49], [53]. The persistence of aquatic fauna within pool beds following surface water loss has been well documented [43], [50], [52], [55]–[62], but few details exist about the terrestrial invaders. Boulton and Suter [49] noted the appearance of a “summer clean-up crew” following surface water loss in a southern Australian intermittent stream bed – it consisted of carabid and hydraenid beetles, lycosid spiders, ants and terrestrial amphipods. The appearance of a similar suite of taxa following surface water disappearance has been recorded elsewhere [43]–[47], [53], [54], [63], [64]. Species richness and abundance appear to decrease following surface water loss, suggesting that the extinction of aquatic fauna occurs more rapidly than the colonization of terrestrial fauna, although previous studies focused mainly on the aquatic fauna and so likely missed a significant component of the community [43], [46], [49], [52]. Little is known about the temporal extent of the moist phase, and while the moisture dynamics within any particular pool bed will vary according to its specific environmental characteristics [62], it is often assumed that water loss is complete and the substrate is totally dry within a few days [43], [46], [49], [52].

There are two possible scenarios for the dynamics of the faunal assemblages within intermittent pool beds following surface water loss. If the terrestrial colonists of the dry pool bed are riparian species, as often implied within the literature [43]–[47], [49], [53], then after the moist phase the community should resemble that within surrounding terrestrial habitat. This implies that intermittent pools are islands of aquatic habitat that periodically appear within the permanent terrestrial environment. Alternatively, if dry intermittent pool beds are colonized by a fauna distinct from surrounding permanently terrestrial habitats, or if the dry phase retains substantial numbers of active aquatic species, then dry pool beds could remain ecologically distinct from the surrounding terrestrial environment until subsequent refilling [23]. If this is true, then intermittent pool beds are cyclic habitats with distinctive species assemblages in both the aquatic and terrestrial phases.

Several lines of evidence suggest that the transitional period from aquatic to terrestrial phases supports a distinct fauna, and that even following full drying, the faunal assemblages remain distinct from those of the surrounding terrestrial habitat. First, a comprehensive study of terrestrial invertebrates within dry river beds and associated riparian zones in Australian and Italian rivers concluded that some fauna are unique to dry stream beds [23]. Second, dry pool beds contain dormant desiccation-resistant stages of aquatic fauna [46], [48], [52], [65], [66] that are not found within the surrounding terrestrial habitat; these may be an important food resource for terrestrial colonists [43]. Third, dry intermittent pool beds often remain distinct from the surrounding terrestrial environment [23]. For example, the topography of pool beds is different from the surrounding environment, this often causes them to collect wind- and water-borne organic debris. Also, drying of the pool bed can occur over protracted periods, during which time the habitat is no longer aquatic, but remains non-terrestrial; and the nature of the leaf litter layer tends to differ from that within surrounding environments, since leaves decay differently when immersed, and previously immersed litter includes dried aquatic detritus and sometimes mats of dried algae [67].

In this study we explored macroinvertebrate community dynamics during the transition from aquatic to terrestrial habitat within a series of natural pool beds, to determine whether and for how long the community within dry pool beds was distinct from that within surrounding permanently terrestrial habitat. Specifically, we monitored and compared macroinvertebrate communities within leaf packs that i) remained wet, ii) underwent drying (i.e., started wet and then dried) and iii) remained dry. We focused on litter fauna because leaf packs occur within intermittent pool beds throughout the year [60] are food and microhabitat for many aquatic and terrestrial macroinvertebrates [68]–[74], and are easily manipulated. Finding that the assemblages of macroinvertebrates inhabiting previously immersed leaf packs were distinct from those of permanently aquatic and permanently terrestrial leaf packs would support the emerging idea [23] that intermittent pool beds are cyclic habitats in which the terrestrial stage, as well as the aquatic stage, is distinct from surrounding habitats. In either case, studying intermittent pool beds as cyclic habitats may yield insight into a variety of ecological processes, such as the colonization and extinction dynamics of species assemblages and the transfer of energy across spatial and temporal habitat boundaries.

## Materials and Methods

### Study site

The study was conducted in Goondaloo Creek, a second-order stream located in the foothills of the Mount Stuart Range, Townsville, Queensland, Australia (19°20′ S, 146°47′ E). No specific permissions were required to undertake field research at this site because it was university property. Within the stretch of stream bed where experiments were undertaken the substrate is composed of granite bedrock, partly covered by patches of sand, pebbles and boulders [75]; a detailed description of the site and study pools is given by Smith and Pearson [60]. Goondaloo Creek normally flows for several weeks in the wet season, then dries to a series of pools, which remain inundated for several months before drying. Our study commenced at the beginning of the dry season, early April, when the stream bed contained numerous intermittent pools ranging in size from <1.0 m diameter and a few cm deep to approximately 8 m wide and>1.5 m deep. A diverse community of macroinvertebrates typically occupies these pools [60]. No fish were observed in our study pools, nor have we ever observed any this far up Goondaloo Creek over 15 years of study.

### Experimental design

The experiment was replicated once in each of six spatial blocks. Each block consisted of a natural intermittent pool and its surrounding terrestrial environment. All pools were located within a 100 m stretch of stream bed, were similar in size at the start of the study (20–34 m^2^ surface area), and were separated by 9–37 m of dry stream bed. Four leaf packs (representing different treatments) were allocated to each replicate block: three packs were immersed in the pool at similar depths (∼15 cm) at ∼35 cm intervals, while the fourth pack was placed on the surrounding dry substrate ∼1 m from the water's edge (the *Terrestrial* treatment). Of the three leaf packs immersed in the pool, two ‘transition’ packs were very carefully and slowly repositioned to the pool margins and allowed to dry (*Transition I* and *Transition II* treatments – see below) while the last remained in the bottom of the pool and was exposed to the same environmental conditions as natural immersed leaf packs (the *Aquatic* treatment). Leaves and associated fauna were sampled from each pack 10 times over four months (see below).

### Leaf packs

Recently abscised and freshly picked leaves from a variety of common tree and shrub species were used to construct the leaf packs; approximately 20% of leaves were fresh. All leaves were collected in March 1998 from the study streambed or from adjacent riparian vegetation. Fresh leaves were collected from plant species in approximate proportion to their occurrence in the collected abscised leaves. Approximately three-quarters of these leaves were conditioned by immersion in rainwater in one of three 900 L plastic water tanks, which were placed outside and covered with plastic mesh (1 mm) to minimize aerial colonization by macroinvertebrates (aerial colonization of aquatic leaf packs occurs naturally, and so it was not essential that every colonization event during this conditioning phase was prevented). Leaves were immersed in the tanks for 3 days to remove terrestrial biota, to leach soluble compounds, and to allow colonization by micro-organisms [68]. Leaching reduces levels of tannins and other soluble compounds in the leaves, which might have polluted the pools if large quantities of unconditioned leaves were introduced at once. Each day, water within the tanks was replaced and samples of leaves were moved among the three tanks to minimize any effects of leaching history. The 24 experimental leaf packs (4 treatments × 6 replicates) were constructed by filling a nylon mesh bag (45 cm diameter × 90 cm long; 5 cm mesh) with leaves. To construct *Aquatic* and *Transition I* and *II* packs each handful of leaves was chosen haphazardly from one of the three tanks. For *Terrestrial* leaf packs we used unconditioned leaves. The assembled packs appeared similar in size and shape to natural leaf packs.

### Sampling and sample processing

All leaf packs were placed in or adjacent to the pool beds on 3 April 1998 (Day 0). Each pack was sampled on Days 15, 34, 53, 56, 64, 79, 93, 96, 104 and 119. Samples were taken more frequently after each drying treatment so that short-term changes in faunal composition could be detected. *Transition I* leaf packs were repositioned after sampling on Day 53; *Transition II* leaf packs after sampling on Day 93. Repositioning involved carefully shifting the leaf pack to the pool margin until at least half of the pack was out of the water. As pools were small and packs were positioned close to the water's edge, packs were moved less than 30 cm. All packs in all treatments were disturbed in a manner similar to the packs undergoing transition, to control for the disturbance and to allow minor relocation of immersed leaf packs to ensure they remained inundated as water levels declined. An out-of-season flood in late August 1998 terminated the study before any pools dried completely.

On each sampling occasion we removed a single grab of leaves (29.63±0.69 [mean ± SE] g dry weight coarse particulate organic matter, CPOM, see below) from the middle of each of the 24 leaf packs using a small aquarium net (15 cm×15 cm; 200 µm mesh) and immediately placed the contents into 70% ethanol. A similar quantity of leaves was gently added to each pack to replace those removed by sampling and by decomposition, which ensured that leaf pack size remained relatively constant throughout the experiment. This procedure approximated natural replenishment of leaf packs within the stream bed [60].

On each sampling date we recorded the maximum depth of each pool. We also estimated the moisture content of each leaf pack in the field, assigning values from 5 (fully aquatic) to 1 (dry). We calibrated these estimates by placing oven-dried filter paper into each of the leaf packs on Days 93, 96, 104 and 119. On the next sampling date these papers were removed and placed into an oven-dried airtight glass jar. We weighed this assembly, then opened the jar and dried the filter paper and jar for two days at 60°C and reweighed them. We used the weight of water lost as an index of the moisture content of each leaf pack, and compared it with the initial estimate. A correlation analysis indicated a high correlation between our estimates and the filter paper-based index (r^2^ = 0.934; P<0.001), validating our estimation technique; therefore, we present only the field moisture estimates as these are available for more sampling dates.

In the laboratory, individual leaves were washed over a sieve stack to remove macroinvertebrates. Animals retained in a 1 mm sieve were identified as far as possible, using published keys. This meant that the taxonomic level to which individuals were identified varied among groups, but most were identified to family level or lower, and some taxa with distinct developmental phases were divided into more than one trophic taxon (Table S1). These data were used to estimate abundance for each taxon. Remaining leaf material (CPOM, >1 mm) was dried for three days at 60°C before being weighed.

### Data analysis

We standardized counts of animals to numbers per 10 g dry weight of CPOM to account for small variations in the size of individual samples. We performed all statistical tests at two-tailed α = 0.05. Multivariate analyses were undertaken using randomization MANOVA tests [76] in which we used 10,000 random reassignments of the log(n+1) transformed abundance data (standardized to Euclidean distance  = 0 by taxon) to generate an empirical distribution of expected values of the test statistic. The observed value was then compared to this distribution to assess significance. Tests were only considered significant when the upper 95% binomial confidence limit for the P-value obtained was <0.05 (CYTEL Software Corporation 1999); it is these conservative P-values that we report in our results. The multivariate test statistic we used was the sum of the Euclidean distances between vectors of variables standardized to mean 0, mean absolute deviation 1, and their treatment centroids [76], [77]. Tests were carried out using a custom-written computer program [78].

Our hypothesis tests were done in a hierarchical manner. Using a single randomisation test we initially tested the overall hypothesis that the time series of log(n+1) transformed abundance data, standardisedstandardized to Euclidean distance  = 0 by taxon, differed among treatments. We treated the data for each replicate of each treatment as a 1020-element vector (102 taxa, each counted 10 times) and used a randomization test to test whether the mean vectors for all treatments were identical. Because this test indicated that there were treatment effects, we performed 10 separate randomization tests, one for each time, to test for treatment effects separately on each sampling date. The significance levels of these tests were adjusted for multiple comparisons using sequential Bonferroni adjustments [79]. The results of all the tests were significant, so we performed pair-wise comparisons among the responses to all treatments at each time. We performed these comparisons at significance levels adjusted to preserve the overall Type I error rate for the suite of comparisons within each time at α = 0.05 using sequential Bonferroni adjustments.

Following these multivariate analyses we used principal component analysis (PCA) to reduce the dimensionality of our data and simplify the illustration of temporal and spatial patterns. We used PCA instead of other ordination techniques (e.g., multidimensional scaling) in order to retain the original multivariate structure of the data. We applied PCA to the square root of the mean count of each taxon (over the six replicates) in each treatment at each time. The square-root transformation was applied to normalize the distributions of the count data. The transformed data were standardized to Z-scores for each variable prior to analysis to prevent abundant taxa from dominating the results. Duplicate PCA's were run on the complete data set and on a data set that excluded *Segnitila* sp. (by far the most common taxa recorded in our study, Table S1). Results of the analyses with and without *Segnitila* were very similar in absolute patterns, so only the results of the analysis of the complete data set are shown.

## Results and Discussion

### The physical environment

**Figure 1:**
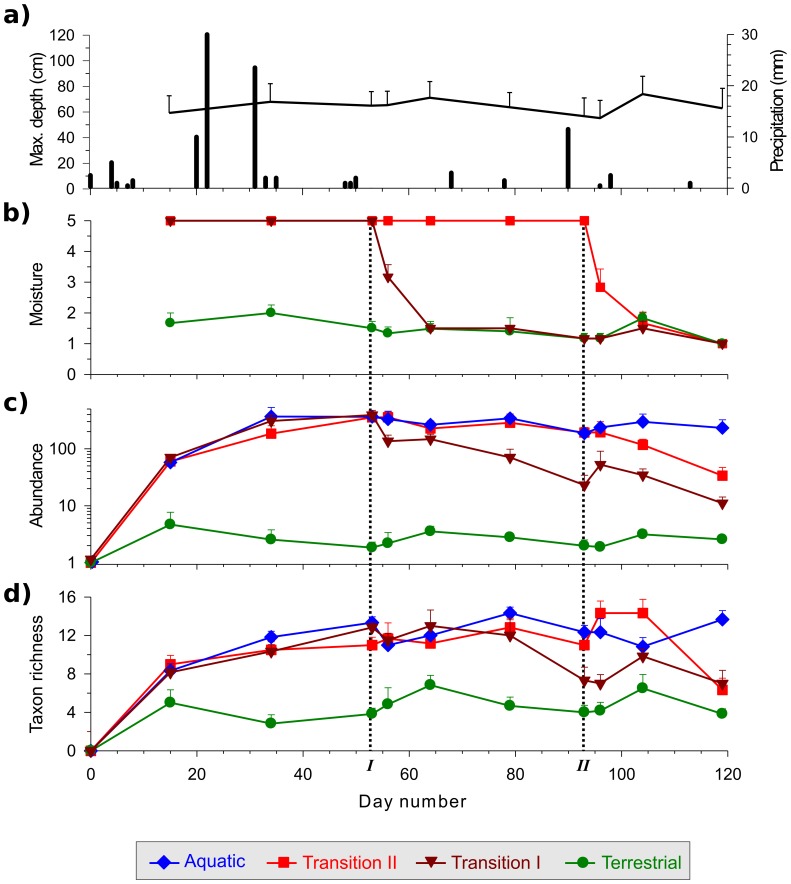
Changes in a) mean maximum pool depth (line) and total daily precipitation (bars); b) mean moisture level of leaf packs; c) abundance of individuals (standardized to number per 10 g dry weight leaf litter); and d) taxon richness (standardized to value per 10 g dry weight leaf litter) over the study. Vertical black dotted lines indicate when each leaf pack transition was undertaken (i.e., *Transition I* and *Transition II*, see Materials and Methods for details). Error bars are +1 SE.

Unseasonal rainfall at the end of the dry season, and the early termination of our field experiment due to storm damage, meant that the depth of all six pools remained relatively constant throughout the study (Figure 1a). The moisture content of *Transition I* and *II* leaf packs decreased to that found in *Terrestrial* packs within 11 days (Figure 1b), meaning that ecological patterns after this time were not caused by moisture levels being higher in dried leaf packs. The convergence in moisture content may have taken substantially less than 11 days, since no data were collected between 3 and 11 days following drying. Although the time to convergence between drying leaf packs and permanently terrestrial leaf packs was short, it is still possible that the ability to colonize during the transitional phase may confer an advantage over later colonists. Such ‘priority effects’ are known to occur in intermittent pool beds when they are aquatic, as well as in other habitats [6], [80]–[85], but their role in structuring the dry pool bed community is unknown.

### Community patterns

A taxonomically and ecologically diverse suite of species was found in and around the intermittent pool beds throughout this study, with a total of 83,419 individuals from 102 taxa recorded (Table S1). Common taxa included gastropods (specifically a single genus of snail, *Segnitila* sp.), many orders of insects, arachnids, and a few frogs and tadpoles (Table S1).

**Figure 2:**
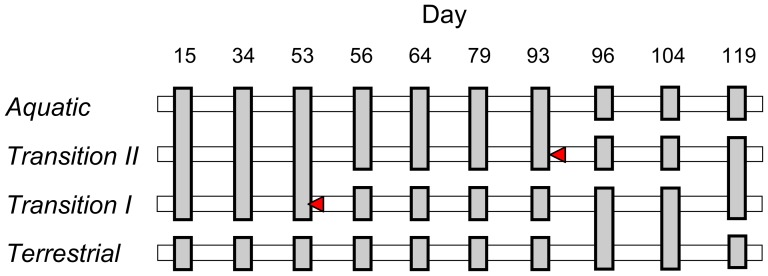
Diagrammatic representation of the results of sequential Bonferroni-adjusted multiple comparisons following multivariate randomization MANOVA tests for treatment differences on each date (see **Table 1**). Red arrowheads indicate when leaf packs underwent habitat transition (see Materials and Methods for details). Connected grey vertical bars indicate treatments that were not significantly different, detached grey bars indicate treatments that were significantly different.

Our initial randomization test revealed that the 1020-element vectors of taxon abundances over time differed among the four treatments, revealing that at least some of the four leaf pack assemblages differed on at least some of the sampling dates (Table 1). Subsequent sequential Bonferroni-adjusted pairwise comparisons revealed how the combined data on taxon abundances and temporal patterns differed among treatments (Table 1, Figure 2). On all ten sampling dates macroinvertebrate assemblage structure in *Aquatic* leaf packs was significantly different from that in *Terrestrial* packs (Table 1, Figure 2). As expected, the assemblages of the *Aquatic, Transition I*, and *Transition II* leaf packs did not differ significantly before the first experimental habitat transition (day 53), when they were all still immersed. Three days after the *Transition I* leaf packs started drying (day 56), their assemblage structure was significantly different from that within both *Aquatic* and *Terrestrial* leaf packs. The *Transition I* assemblages remained different from *Aquatic* ones, and were significantly different from the *Terrestrial* assemblages, on days 64, 79, 104, and 119, indicating that, although they were not significantly different at approximately 40–43 days after drying (day 93–96), the assemblages of *Transition I* and permanently terrestrial leaf packs differed for at least 66 days after surface water loss. Following drying, *Transition II* leaf packs followed similar patterns to *Transition I* packs: they diverged significantly from the *Aquatic* assemblage by three days post-drying (day 96), and remained distinct from the *Terrestrial* assemblage for the remainder of the study. On the last sampling date (day 119), faunal assemblages in the *Transition I* and *Transition II* packs did not differ significantly, but both differed significantly from assemblages both in the *Aquatic* and *Terrestrial* packs.

**Table 1:** Results of multiple randomization MANOVA's to determine treatment effects on community composition.

	All	Aq–Ter	Aq–T I	Aq–T II	T II–T I	T II–Ter	T I–Ter
All times	<0.001	0.003	0.002	0.003	0.001	0.003	0.002
Day 15	<0.001	0.002	NS	NS	NS	0.003	0.003
Day 34	<0.001	0.003	NS	NS	NS	0.001	0.002
Day 53	<0.001	0.003	NS	NS	NS	0.003	0.002
*Transition I*							
Day 56	<0.001	0.002	0.002	NS	0.003	0.002	0.002
Day 64	<0.001	0.003	0.002	NS	0.002	0.003	0.002
Day 79	<0.001	0.002	0.003	NS	0.003	0.003	0.004
Day 93	<0.001	0.003	0.002	NS	0.004	0.003	0.010
*Transition II*							
Day 96	<0.001	0.002	0.004	0.007	0.004	0.001	NS
Day 104	<0.001	0.004	0.004	0.003	0.018	0.001	NS
Day 119	<0.001	0.002	0.002	0.003	NS	0.027	0.033

Number indicates P value with upper 95% confidence limit; NS indicates no significant difference between treatments. Aq  =  *Aquatic*, T I  =  *Transition I*, T II  =  *Transition II*, Ter  =  *Terrestrial*. Only P values that were significant following sequential Bonferroni adjustment are shown (see Materials and Methods).

Wet leaf packs and dry leaf packs that had previously been wet always supported more taxa and higher abundances than *Terrestrial* leaf packs (Figure 1c–d). In contrast to previous work [22], there were no major shifts in taxon richness or abundance immediately following drying, despite the habitat undergoing major environmental transition from wet to dry. This suggests that colonization by terrestrial individuals compensated for the disappearance of remnant aquatic fauna (see below). In fact, although over the longer term there was a general decrease in both richness and abundance towards levels found within *Terrestrial* packs, taxon richness increased immediately following drying in recently emersed leaf packs. This richer community appears to be maintained for up to 11 days after surface water loss (Figure 1d). At the last sample date the numbers of taxa and abundance in *Transition I* and *Transition II* leaf pack assemblages were similar, and were intermediate between the higher values found in *Aquatic* packs and the lower values found within *Terrestrial* packs (Figure 1c–d). It is noteworthy that patterns in abundance and richness were largely consistent between the two transition leaf packs, despite their drying being separated by 40 days. This suggests that the patterns we have documented result directly from pool drying and are not simply seasonal effects. The transition from aquatic to terrestrial habitat within the pool bed appears to initiate a predictable shift in community structure, despite its temporal unpredictability.

Small rises in the number of taxa occurred in all emersed leaf packs (including *Terrestrial, Transition I* and *Transition II* packs that had undergone drying) approximately 11 days after each transition (Figure 1d). For example, following drying, *Transition I* packs experienced peaks in taxon richness on day 64 (11 days after emersion) and also on day 104 (11 days after emersion of *Transition II* packs). This result was unexpected, because if the surrounding terrestrial habitat acts as a source of colonists for dry pool beds [43], [46], [52], [55] then taxa richness within newly dry leaf packs should decrease immediately after emersion, and should then approach the lower richness of permanently terrestrial leaf packs as individuals migrate in from the terrestrial packs. It seems plausible that a drying pool bed attracts taxa that specialize on that habitat, or generalist taxa that scavenge on the newly-available terrestrial resources; they may also temporarily colonize nearby permanently terrestrial habitat [25]. This ‘spill-over’ effect could explain why the *Terrestrial* and *Transition I* treatments were not significantly different on days 96 and day 104 when overall differences in assemblage structure were found (Table 1, Figure 2). If this is true, the similarity of these two assemblages is a result of the convergence of the *Terrestrial* assemblage structure towards that of the *Transition I* assemblage, rather than vice-versa (see below, Table S1). An alternative mechanism that could explain the simultaneous colonization of all emersed packs by new taxa could be the seasonal appearance of taxa; however, this seems unlikely as the only two peaks we observed in all dry packs (including both *Terrestrial* and emersed *Transition* packs) both occurred around 11 days after a transition occurred.

**Figure 3:**
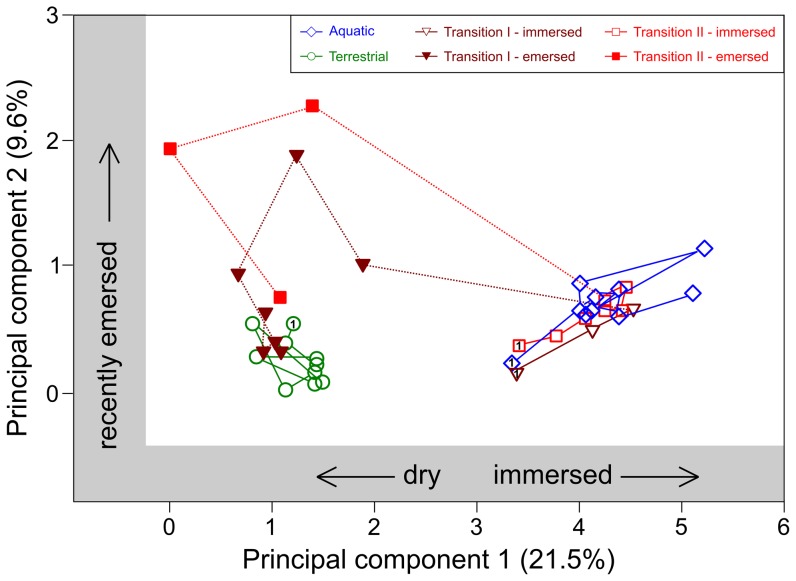
Successional trajectories of faunal communities (mean across all replicates of PC scores based on square-root transformed abundance of each taxon) of the four experimental treatments as revealed by changes on the first two axes of a principal components analysis. Sequential data points for each treatment are joined by lines. Dotted lines show temporal sequences for Transition leaf packs. Symbols with number ‘1’ inset indicate the mean of the first samples for each treatment (i.e., Day 15). Axis percentages represent percent of total variance explained by that axis.

Patterns of change in assemblage structure and composition over time are displayed in Figure 3, which shows the mean scores on the first two PC axes for the six replicates of each treatment. Scores on the first principal component increased with the prevalence of taxa in the aquatic phase of the habitat and decreased with higher levels of taxa and abundance in the dry environment. The second principal component increased with taxa in drying leaf packs and decreased with increases in taxa found in either immersed or *Terrestrial* leaf packs. The communities of the *Aquatic* and *Terrestrial* treatments remained distinct, occupying different regions of community space throughout the experiment (Figure 3). When removed from the pool, leaf pack assemblages diverged from *Aquatic* communities but did not immediately converge with those within surrounding terrestrial leaf packs. Assemblages in *Transition I* and *Transition II* leaf packs followed similar successional trajectories after emersion, despite their drying dates being separated by 40 days. Although the final composition and structure of transition assemblages was close to that of *Terrestrial* assemblages, they still differed from them on the last sampling date (Table 1; Figure 2, Figure 3).

### Specific faunal groups

**Figure 4:**
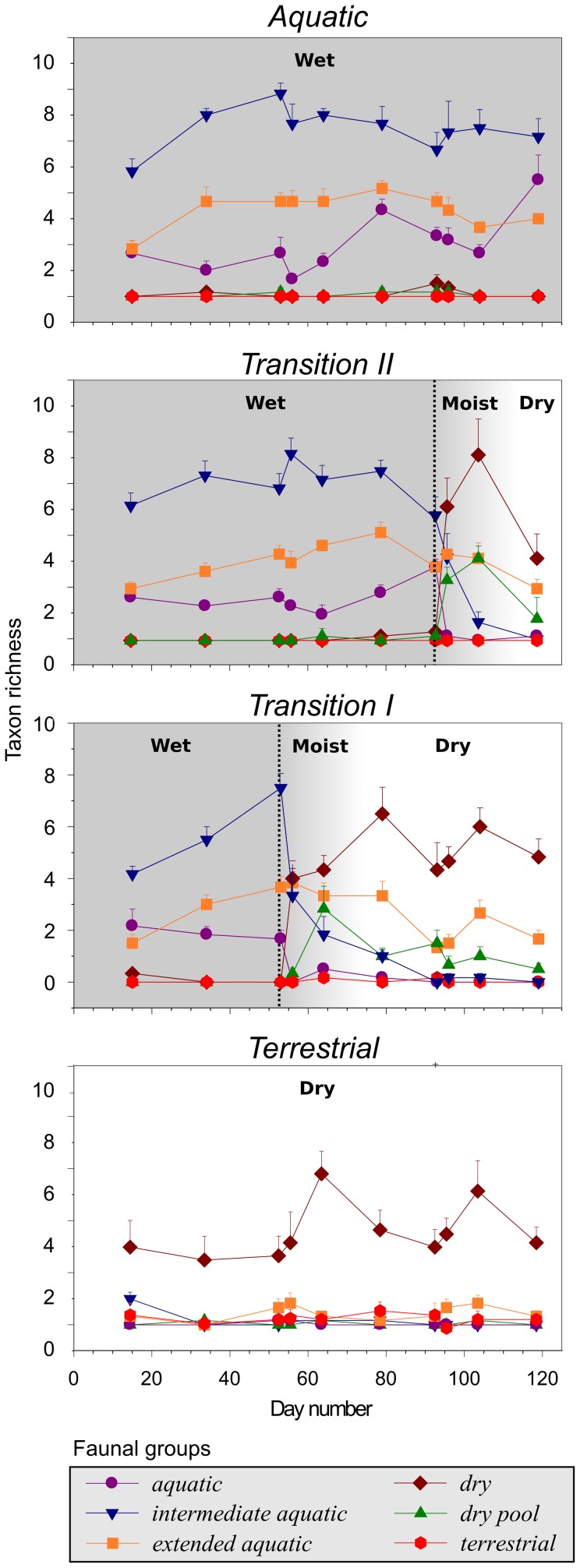
Temporal changes in taxon richness in the six faunal groups in each leaf pack treatment (+1 SE). Vertical black dotted lines indicate when each leaf pack transition was undertaken (i.e., *Transition I* and *Transition II*, see Materials and Methods for details). Panel shading denotes different habitat phases: grey  =  wet, light grey  =  moist, and white  =  dry.

**Figure 5:**
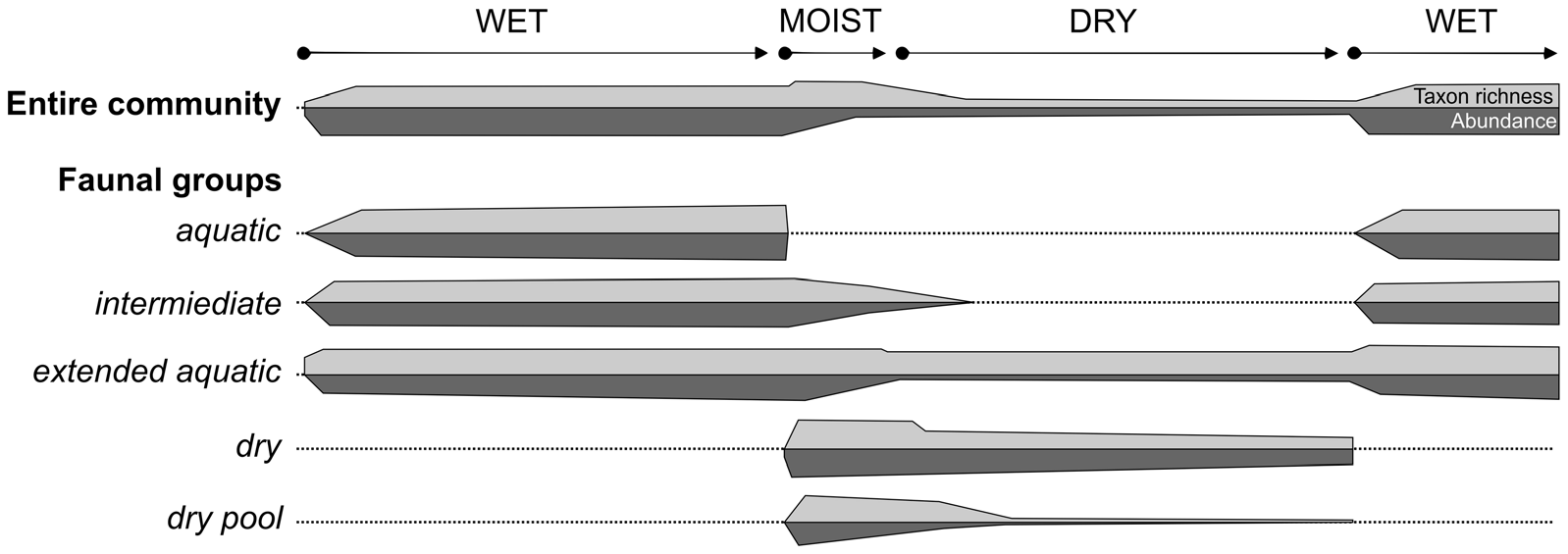
Hypothesized dynamics of the entire pool bed assemblage and individual faunal groups during the annual wet-dry cycle of an intermittent pool bed. Light horizontal bars represent taxon richness and dark horizontal bars represent abundance. The thickness of the bars represents numbers relative to the maximum recorded throughout the year within that category.

Two main processes determined the dynamics of pool bed communities during drying: loss of pool fauna and colonization by terrestrial fauna. To provide a clearer picture of this dynamic interplay between extinction and colonization we grouped taxa that showed similar patterns of occurrence (abundance and presence–absence) within leaf packs of different treatment and age (i.e., time since emersion). All taxa fit into one of six broad patterns of occurrence (Figure 4, Figure 5, Table S1). Three of these faunal groups were comprised of species found primarily when the pool bed was aquatic, two were comprised of species found in formerly immersed leaf packs, and one was limited to permanently terrestrial leaf packs. The definitions and patterns of occurrence of these groups are discussed in detail below.

The three aquatic faunal groups we identified were: ‘aquatic’, which disappeared from leaf packs immediately following drying (e.g., Dytiscidae, Hydrophilidae, and Gyrinidae larvae and adults, Corixidae, Hydracarina, and larval Culicidae and Tabanidae), ‘intermediate aquatic’, which persisted in the drying leaf packs for one to two weeks following emersion (e.g., Ostracoda, Ephemeroptera and Odonata larvae, and Chironomidae and Ceratopogonidae), and ‘extended aquatic’, which consisted of taxa whose abundances were heavily affected by drying, but whose presence was not (e.g., Planorbidae and Physidae snails, and Psephenidae larvae) (Figure 4, Figure 5, Table S1). Similar types of species, and patterns of occurrence and abundance, have been recorded in other pool bed systems across the globe, and represent the different ways in which pool fauna cope with the temporary nature of their habitat [46], [55], [61], [62], [65]. For example, many taxa within the ‘aquatic’ group cope with pool drying by moving from the habitat, either as they are (e.g., adult Coleoptera) or by undergoing transformation into a terrestrial form (e.g., larvae of Dipteran, Ephemeroptera, and Coleoptera) [46]. In contrast, ‘extended aquatic’ taxa have desiccation-resistant stages that enable them to survive the dry phase of the habitat *in situ* (e.g., *Segnitila* - Gastropoda) [43], [46], [50], [52], [55]–[62], [65]. Therefore, many remnant aquatic-adapted fauna that persist in the dry pool bed are likely to be in a diapause state where they are not actively feeding. However, such taxa are not necessarily ecologically inactive components of the community, as they are likely still an important food resource for other actively feeding taxa [43]–[47], [49]. These aquatic taxa, and any terrestrial taxa that do the opposite (see below), would have altered ecological roles in each of the different habitat phases.

The three primarily terrestrial faunal groups we identified included two groups that occurred within formerly immersed leaf packs (both the ‘dry’ and ‘dry pool’ fauna appeared following emersion, but the ‘dry pool’ fauna appeared somewhat more slowly and did not colonize permanently terrestrial leaf packs, while the ‘dry’ fauna persisted well into the terrestrial phase and also colonized permanently terrestrial leaf packs) and a third, the ‘terrestrial’ fauna, that only occurred within leaf packs that remained permanently dry (i.e., *Terrestrial* packs) (Figure 4, Figure 5). This ‘terrestrial’ group included species that are characteristic of terrestrial leaf packs throughout the globe, such as Coleoptera, Formicidae, and adult Diptera (Table S1). In general, the ‘terrestrial’ fauna was patchy in distribution and usually present in low numbers (Table S1).

The ‘dry’ fauna, which was common to drying (*Transition I* and *II*) and permanently dry (*Terrestrial*) leaf packs, consisted of a diverse group of invertebrates that colonized drying leaf packs from the surrounding terrestrial habitat. Principal taxa included Isopoda, Collembola, Orthoptera, Psocoptera, Lygaeidae, Coleoptera (particularly from the families Anthicidae, Elateridae, Staphylinidae and Psephenidae), Psychodidae and Lepidoptera larvae, Formicidae and Arachnida (Table S1). No single taxon was numerically dominant and none was extremely rare (Table S1). Many of these taxa have been observed in drying and dry pool beds previously [22], [43]–[46], [49], [52], [54], [86]. Once the ‘dry’ fauna colonized, many taxa persisted in the previously immersed leaf packs for the remainder of the study (Figure 4b–c, Figure 5). Generally, more ‘dry’ taxa were found within previously immersed leaf packs than in *Terrestrial* packs (Figure 4b–d), suggesting that although ‘dry’ taxa are capable of inhabiting a variety of dry leaf pack types, the habitat provided by previously immersed leaf packs is of higher quality than within permanently *Terrestrial* packs. Hence, dry intermittent pool beds may be important for the maintenance of ‘dry’ taxa.

Our ‘dry pool’ fauna was comprised of taxa that occurred almost entirely within dry leaf packs that had previously been immersed (Table S1). This faunal group contained more than twice as many taxa as the terrestrial group, and while some taxa were recorded in abundances similar to terrestrial taxa the majority were recorded in multiple leaf packs (Table S1). Dominant taxa included Chilopoda, Psylloidiae and Peloridae bugs, Coleoptera (particularly Hydraenidae and Carabidae), Mecoptera, Thysanoptera, and Ceratapogonidae larvae from the subfamily Forcipomyiinae (Table S1). Because we did not identify individuals to species we do not know where, or if, these taxa have been previously recorded. In both *Transition I* and *Transition II* leaf packs, taxon richness of our ‘dry pool bed’ fauna peaked about 11 days following emersion, and while there was a tendency for both richness and abundance to decrease over time, previously immersed leaf packs always contained detectable levels of ‘dry pool bed’ fauna (Figure 4b–c, Figure 5). This fauna may represent a suite of species that preferentially occupy dry intermittent pool beds [23]. Although we cannot discount the possibility these species have inundation-resistant forms to survive the wet phase *in situ* [23], [87]–[90], it seems more likely that most survive wetting episodes either by movement into surrounding permanent terrestrial environment, or by movement from one dry pool bed to another as individual pool beds fill and dry. This hypothesis is supported by our results showing that ‘dry’ fauna colonized emersed packs more quickly than did ‘dry pool’ fauna, which would be expected if the latter had to disperse a greater distance from other surrounding dry pool beds (cf. ∼3 days for ‘dry’ fauna with ∼11 days for the ‘dry pool bed’ fauna) (Figure 4b–c). Also, the majority of ‘dry pool’ fauna are winged [86] and could therefore easily disperse between dry pool beds.

### Dry pool beds remain distinct from the surrounding terrestrial environment

Our results indicate that the macroinvertebrate community within pool beds can remain different from that within the surrounding streambed throughout all their habitat phases [23], including both the aquatic and terrestrial phases. We found that marked differences between the assemblages of previously immersed and permanently *Terrestrial* leaf packs remained 66 days after emersion. In our study, differences between dry pool bed and terrestrial assemblages were caused by i) the appearance of a faunal group that was unique to dry pool beds (the ‘dry pool bed’ taxa), ii) a higher number of taxa that were common to both dry pool beds and the surrounding terrestrial environment (the ‘dry’ taxa), iii) the existence of some remnant aquatic taxa (the ‘extended aquatic’ taxa), and iv) the absence of some taxa that were found within the surrounding terrestrial environment (the ‘terrestrial’ taxa).

A variety of factors could result in dry pool beds supporting a different macroinvertebrate community from that within the surrounding terrestrial environment [23], [53]. For example, leaf packs in dry pool beds could remain wetter, even after surface water loss, thus supporting a more complex community. However, in our study, moisture levels of leaf packs removed from the pool environment matched those of fully *Terrestrial* packs within 11 days, so differences after this date could not have been caused by higher water availability. Another factor is that leaves within dry pool beds have been previously immersed, making them more palatable for detritivorous macro-consumers than those that have never been immersed [21], [53], [54]. A different, or at least enriched, basal consumer group within dry pool beds (feeding on the conditioned leaves) would also likely support a different group of higher consumers from those found in surrounding terrestrial habitat. Another factor could be that remnant aquatic individuals provide a rich supply of food for invading scavengers and predators. This is certainly true for a few days following surface water loss [23], [43]–[46], [49], [52]–[54], but these accounts are not sufficiently resolved taxonomically to allow detailed trophic analysis.

We found ecological differences between ‘dry pool bed’ and ‘terrestrial’ macroinvertebrate assemblages for 66 days following surface water loss, and our results may understate the differences. *Terrestrial* leaf packs were placed within the stream bed just below maximum flood height, not within the true permanently terrestrial riparian zone, and thus may have been colonized initially by fauna that inhabit dry pool beds, decreasing the differences we found. In addition, in natural systems environmental changes prior to water loss can act as a cue to some aquatic species that pool drying is imminent [46], [52]. In our study there were no such cues as leaf packs were removed from the pool beds when they still had water in them, so fauna may not have reacted as they would during and following natural drying; this could affect the transition in a variety of ways.

## Conclusion

We have demonstrated that pool drying is characterized by extinctions and colonizations of distinct faunal groups as the habitat shifts from aquatic to terrestrial (Figure 5). The dynamics of the macroinvertebrate community during drying represented a rapid changeover from an aquatic to a terrestrial community, and were driven by different responses of distinct faunal groups with differing adaptations to intermittency. Our data suggest that drying of the leaf pack initiated invasion by fauna from surrounding permanently terrestrial habitat, and by a suite of taxa that are unique to dry pool beds. They also suggest that at least some of these taxa spilled over to surrounding leaf packs, even those that had never been immersed, increasing the taxon richness of terrestrial habitat adjacent to intermittent aquatic habitat. Our data support the hypothesis that dry pool beds are ecologically distinct from surrounding permanently terrestrial habitat, making efforts to conserve intermittent pool bed habitats even more pressing in the face of multiple anthropogenic stressors [21], [23]–[25], [91].

The value of intermittent pool beds as model habitats increases significantly with the recognition they possess unique intermittent aquatic and terrestrial habitat phases. These systems provide a unique opportunity to compare natural aquatic and terrestrial communities, and the dynamics of a natural shift between the two, within the same physical space, separated by only a few months. Intermittent pool beds are novel because they not only have a spatial aquatic-terrestrial ecotone (at the margins of the pool bed when it is full), but also a ‘temporal ecotone’ that is created by the repeated filling and drying of the pool bed [21], [22], [24], [48], [86]. We did not measure the temporal transfer of energy and materials between phases, but the interlinked nature of the different faunal groups we observed suggests that these temporal linkages are strong. Our results suggest that examination of single habitat phases in isolation represent an over-simplified view of the ecology of these systems. Intermittent pool beds are more than temporary aquatic habitats: they are truly cyclic habitats that support distinct terrestrial and aquatic species assemblages.

## Supporting Information

Table S1 **Abundance of individual taxa within each faunal group.**
(DOCX)
