# Supplementary material for: Intermittent Pool Beds Are Permanent Cyclic Habitats with Distinct Wet, Moist and Dry Phases
Source: PLoS One. 2014 Sep 22;9(9):e108203. doi: 10.1371/journal.pone.0108203 (PMC4171517; doi:10.1371/journal.pone.0108203)
Supplement: Table S1 — Abundance of individual taxa within each faunal group. (DOCX) [file pone.0108203.s001.docx]

Supporting Information

**Intermittent pool beds are permanent cyclic habitats with distinct wet, moist and dry phases**

Anthony I. Dell*, Ross A. Alford and Richard G. Pearson

*Corresponding author: [adell@gwdg.de](mailto:adell@gwdg.de)

**Table S1. Abundance of individual taxa within each faunal group.** Column headings denote treatments (see main text). Values represent abundance per 10 g dry CPOM averaged for all six replicates. Values in parentheses represent proportion of leaf packs of each treatment (*Terrestrial* = 10; *Transition I* wet = 3; *Transition I* dry = 7; *Transition II* wet = 7; *Transition II* dry = 3; *Aquatic* = 10) inhabited by each taxon (all replicate packs included). - indicates absence; l, larva; a, adult; p, pupa; t, tadpole; m, metamorph.

|  |  |  |  |  | Transition I | | Transition II | |  |
| --- | --- | --- | --- | --- | --- | --- | --- | --- | --- |
|  |  |  |  | Terrestrial | (wet) | (dry) | (wet) | (dry) | Aquatic |
| **Aquatic** | | |  |  |  |  |  |  |  |
|  | Gastropoda | Ancylidae | *Ferrissia* sp. | -(-) | -(-) | -(-) | -(-) | -(-) | 0.4(10) |
|  | Cnidaria |  | *Hydra* sp. | -(-) | -(-) | -(-) | -(-) | -(-) | 0.4(10) |
|  | Plecoptera | Gripopterygidae (l) |  | -(-) | -(-) | -(-) | -(-) | -(-) | 0.5(10) |
|  | Coleoptera | Dytiscidae | Unidentified (l) | -(-) | 5.5(100) | -(-) | 9.8(100) | -(-) | 15.7(90) |
|  |  |  | *Copelatus* sp. (a) | -(-) | 8.7(100) | -(-) | 59.4(100) | -(-) | 49.7(90) |
|  |  |  | *Hydaticus* sp. (a) | -(-) | -(-) | -(-) | 0.2(14) | -(-) | 0.8(20) |
|  |  |  | *Tiporus* sp. (a) | -(-) | 0.4(33) | -(-) | -(-) | -(-) | -(-) |
|  |  |  | *Cybister* sp. (a) | -(-) | -(-) | -(-) | -(-) | -(-) | 0.5(10) |
|  |  |  | *Clypeodytes* sp. (a) | -(-) | -(-) | -(-) | 0.4(14) | -(-) | -(-) |
|  |  | Hydrophilidae | *Enochrus* sp. (a) | -(-) | -(-) | -(-) | 7.9(86) | -(-) | 7.4(50) |
|  |  |  | *Hydrobiomorpha* sp. (a) | -(-) | 3.3(100) | -(-) | 1.3(29) | -(-) | 3.1(60) |
|  |  | Gyrinidae | (a) | -(-) | -(-) | -(-) | -(-) | -(-) | 0.7(30) |
|  |  |  | (l) | -(-) | 4.1(100) | -(-) | 5.7(71) | -(-) | 3.7(50) |
|  |  |  | Noteridae (a) | -(-) | 0.4(33) | -(-) | -(-) | -(-) | -(-) |
|  | Hemiptera | Corixidae |  | -(-) | 1.4(67) | -(-) | 6.0(57) | -(-) | 13.9(80) |
|  |  | Notonectidae |  | -(-) | -(-) | -(-) | 0.6(14) | -(-) | 3.0(50) |
|  |  | Nepidae |  | -(-) | -(-) | -(-) | -(-) | -(-) | 0.7(20) |
|  | Diptera | Unidentified (p) |  | -(-) | -(-) | -(-) | 1.1(29) | -(-) | 1.9(40) |
|  |  | Culicidae (l) |  | -(-) | 0.8(33) | -(-) | 6.7(57) | -(-) | 9.7(50) |
|  |  | Tabanidae (l) |  | -(-) | -(-) | -(-) | 0.4(14) | -(-) | 2.6(50) |
|  |  | Simuliidae (l) |  | -(-) | -(-) | -(-) | -(-) | -(-) | 0.4(10) |
|  |  | Stratiomyidae (l) |  | -(-) | -(-) | -(-) | 0.3(14) | -(-) | -(-) |
|  |  | Ceratopogonidae (a) | | -(-) | -(-) | -(-) | -(-) | -(-) | 0.9(10) |
|  | Trichoptera | Leptoceridae (l) |  | -(-) | -(-) | -(-) | 0.7(29) | -(-) | -(-) |
|  |  | Hydroptilidae (l) |  | -(-) | 0.4(33) | -(-) | -(-) | -(-) | 0.9(10) |
|  | Arachnida | Acarina | Hydracarina | -(-) | -(-) | -(-) | 0.2(14) | -(-) | -(-) |
|  | Anura |  | *Bufo* *marinus* (t) | -(-) | 50.6(33) | -(-) | 28.1(14) | -(-) | 19.2(20) |
|  |  |  | *Litoria* *lesueuri* (t) | -(-) | 1.2(33) | -(-) | 0.5(14) | -(-) | 0.3(10) |
|  |  |  | *Litoria* *lesueuri* (m) | -(-) | 0.7(33) | -(-) | -(-) | -(-) | -(-) |
| **Intermediate aquatic** | | |  |  |  |  |  |  |  |
|  | Annelida | Oligochaeta |  | -(-) | 23.8(100) | 1.2(43) | 35.2(86) | 1.1(33) | 95.7(100) |
|  | Ostracoda | Cyprididae |  | -(-) | 41.7(100) | 9.7(43) | 180.5(100) | 18.2(66) | 229.5(100) |
|  |  | Cytherideidae |  | -(-) | 17.4(100) | 5.6(57) | 58.2(100) | 17.2(66) | 133.8(100) |
|  | Ephemeroptera | Leptophlebiidae (l) |  | 0.6(10) | 119.9(100) | 1.4(29) | 400.9(100) | 1.1(33) | 732.7(100) |
|  |  | Caenidae (l) |  | -(-) | 73.4(100) | 1.4(14) | 106.8(100) | 2.6(33) | 125.6(100) |
|  |  | Baetidae (l) |  | -(-) | 51.1(100) | 0.5(14) | 65.0(100) | -(-) | 83.3(100) |
|  | Odonata | Corduliidae / Libellulidae (l) | | -(-) | 52.7(100) | 0.3(14) | 132.7(100) | 0.2(33) | 219.2(100) |
|  | Coleoptera | Bothrideridae (a) |  | 0.4(10) | -(-) | 1.6(29) | 0.8(14) | 0.8(33) | 2.2(10) |
|  | Diptera | Ephydridae (l) |  | -(-) | -(-) | 0.3(14) | 6.9(29) | 0.3(33) | -(-) |
|  |  | Chironomidae (l) |  | 0.7(10) | 221.8(100) | 5.6(29) | 448.5(100) | 3.3(66) | 605.2(100) |
|  |  | Ceratopogonidae (l) | | -(-) | 2.6(33) | 1.5(14) | 5.5(43) | 0.5(33) | 22.4(80) |
|  |  | Tipulidae Sp. 1 (l) |  | -(-) | -(-) | 0.8(14) | 0.4(14) | 0.7(33) | 1.3(20) |
| **Extended aquatic** | | |  |  |  |  |  |  |  |
|  | Gastropoda | Planorbidae | *Segnitila* sp. | -(-) | 3211.7(100) | 2028.4(100) | 8072.2(100) | 1671.4(100) | 13068.1(100) |
|  |  |  | *Bayardella* sp. | -(-) | 10.9(67) | 12.1(71) | 27.0(100) | 4.6(100) | 68.0(100) |
|  |  |  | *Isidorella* sp. | -(-) | 1.4(67) | 5.4(86) | 4.4(57) | 1.9(66) | 12.1(70) |
|  |  | Physidae | *Physa* *acuta* | -(-) | 75.9(100) | 40.9(71) | 278.7(100) | 198.8(100) | 505.7(100) |
|  | Coleoptera | Psephenidae (l) |  | 2.7(70) | 13.8(100) | 16.1(86) | 22.6(100) | 4.6(100) | 31.5(80) |
| **Dry** | | |  |  |  |  |  |  |  |
|  | Isopoda | Onoscidae |  | 9.1(60) | -(-) | 10.6(71) | -(-) | 3.6(100) | -(-) |
|  | Collembola | Entomobryidae |  | 17.5(90) | -(-) | 24.1(100) | -(-) | 3.5(100) | -(-) |
|  |  | Paronellidae |  | 0.7(20) | -(-) | 0.5(29) | -(-) | -(-) | -(-) |
|  | Blattodea | Blattidae |  | 9.4(80) | -(-) | 7.9(71) | -(-) | 1.9(66) | -(-) |
|  | Orthoptera | Acrididae |  | 1.3(40) | -(-) | 0.8(43) | -(-) | 1.3(33) | -(-) |
|  |  | Gryllidae | Oecanthinae | 0.6(20) | -(-) | 1.8(57) | -(-) | 1.6(66) | -(-) |
|  | Psocoptera | Epipsocidae |  | 7.7(80) | -(-) | 4.3(71) | -(-) | 2.2(100) | -(-) |
|  | Hemiptera | Lygaeoidae | Pyrrhocoridae | 1.6(30) | -(-) | 0.6(29) | -(-) | 0.3(33) | -(-) |
|  | Coleoptera | Anthicidae (a) |  | 2.9(40) | -(-) | 4.1(86) | -(-) | 0.8(33) | -(-) |
|  |  | Passalidae (a) |  | 0.5(20) | -(-) | 0.7(29) | -(-) | 1.8(66) | -(-) |
|  |  | Elateridae (a) |  | 2.6(70) | -(-) | 6.2(86) | -(-) | -(-) | -(-) |
|  |  | Staphylinidae | Oxytelinae (a) | 4.5(60) | -(-) | 1.6(43) | -(-) | 3.1(100) | -(-) |
|  |  |  | Paederinae (a) | 1.0(30) | -(-) | 3.4(57) | -(-) | 1.5(33) | -(-) |
|  |  |  | Tachyporinae (a) | 2.0(30) | -(-) | 1.8(57) | -(-) | 1.7(100) | -(-) |
|  |  | Pspehenidae (a) |  | 3.7(40) | -(-) | 16.7(86) | -(-) | 57.9(100) | 0.7(10) |
|  | Diptera | Psychodidae | (l) | 14.7(20) | 1.0(33) | 1.9(14) | -(-) | 3.0(100) | -(-) |
|  | Lepidoptera |  | (l) | 39.1(90) | -(-) | 20.5(86) | -(-) | 2.8(100) | -(-) |
|  | Hymenoptera | Myrmeciinae | Sp. 1 | 0.7(30) | -(-) | 5.2(86) | -(-) | 16.6(66) | 0.5(10) |
|  |  |  | *Oecophylla* *smaragdina* | 4.6(70) | -(-) | 3.2(71) | 1.5(29) | 4.2(100) | -(-) |
|  |  | Formicinae |  | 0.7(30) | -(-) | 5.2(29) | -(-) | 1.6(66) | -(-) |
|  | Arachnida | Araneae |  | 13.7(100) | -(-) | 11.0(100) | -(-) | 8.4(100) | 0.3(10) |
| **Dry pool bed** | | |  |  |  |  |  |  |  |
|  | Chilopoda | Geophilidae |  | 0.2(10) | -(-) | 5.3(57) | -(-) | 0.3(33) | -(-) |
|  |  | Scolopendridae |  | -(-) | -(-) | -(-) | -(-) | 0.5(66) | -(-) |
|  | Ephemeroptera | Leptophlebiidae (a) |  | -(-) | -(-) | -(-) | -(-) | 0.4(33) | -(-) |
|  | Mantodea | Mantidae | Mantinae | -(-) | -(-) | 0.2(14) | -(-) | -(-) | -(-) |
|  | Hemiptera | Psylloidea |  | -(-) | -(-) | -(-) | -(-) | 1.2(33) | -(-) |
|  |  | Peloridiidae |  | -(-) | -(-) | 0.2(14) | -(-) | 0.4(33) | -(-) |
|  |  | Sp 1. (a) |  | -(-) | -(-) | 0.2(14) | -(-) | -(-) | -(-) |
|  |  | Hebridae |  | -(-) | -(-) | 0.5(14) | -(-) | -(-) | -(-) |
|  | Coleoptera | Sp. 2 (l) |  | -(-) | -(-) | 0.2(14) | -(-) | -(-) | -(-) |
|  |  | Hydraenidae (a) |  | -(-) | -(-) | 5.0(29) | -(-) | -(-) | 0.4(10) |
|  |  | Chrysomelidae (a) |  | -(-) | -(-) | 1.2(29) | -(-) | -(-) | -(-) |
|  |  | Pselaphidae (a) |  | -(-) | -(-) | -(-) | -(-) | 0.4(33) | -(-) |
|  |  | Staphylinidae | Aleocharinae (a) | -(-) | -(-) | 0.4(14) | -(-) | -(-) | -(-) |
|  |  | Carabidae | Sp. 1 (a) | 1.0(10) | -(-) | 9.6(71) | -(-) | 5.9(100) | 0.4(10) |
|  |  |  | Sp. 2 (a) | 0.6(10) | -(-) | 1.1(29) | 0.5(14) | 1.7(100) | -(-) |
|  | Mecoptera | Choristidae | (l) | -(-) | -(-) | 2.4(43) | -(-) | 1.1(66) | 0.5(10) |
|  |  |  | (a) | -(-) | -(-) | -(-) | -(-) | 0.2(33) | -(-) |
|  | Thysanoptera | Phlaeothripidae | *Allothrips* sp. | -(-) | -(-) | 1.0(57) | -(-) | 0.2(33) | -(-) |
|  | Diptera | Heleomyzidae (a) |  | -(-) | -(-) | -(-) | -(-) | 1.2(66) | -(-) |
|  |  | Sciaridae (l) |  | -(-) | -(-) | -(-) | -(-) | 0.8(33) | -(-) |
|  |  | Ceratopogonidae | Forcipomyiinae (l) | -(-) | -(-) | 176.4(43) | 0.5(14) | 13.1(66) | -(-) |
|  |  | Tipulidae Sp. 2 (l) |  | -(-) | -(-) | -(-) | -(-) | 1.7(33) | -(-) |
|  | Lepidoptera (a) |  |  | -(-) | -(-) | 0.5(14) | -(-) | -(-) | -(-) |
|  | Anura |  | *Litoria* *lesueuri* (a) | -(-) | -(-) | 0.6(29) | -(-) | 0.4(33) | -(-) |
| **Terrestrial** | | |  |  |  |  |  |  |  |
|  | Gastropoda | Planorbidae | *Pygmanisus* sp. | 0.3(10) | -(-) | -(-) | -(-) | -(-) | -(-) |
|  | Mantodea | Mantidae | Iridopteryginae | 0.5(20) | -(-) | -(-) | -(-) | -(-) | -(-) |
|  | Hemiptera | Reduviidae | Emesinae | 0.5(20) | -(-) | -(-) | -(-) | -(-) | -(-) |
|  | Coleoptera | Anobiidae (a) |  | 1.4(30) | -(-) | 0.3(14) | -(-) | -(-) | -(-) |
|  |  | Sp. 1 (l) |  | 0.8(20) | -(-) | -(-) | -(-) | -(-) | -(-) |
|  |  | Hydrophilidae |  | 0.3(10) | -(-) | -(-) | -(-) | -(-) | -(-) |
|  |  | Silvanidae | *Gryptamorpha* sp. (a) | 2.3(40) | -(-) | 0.5(14) | -(-) | -(-) | -(-) |
|  | Diptera | Mycetophilidae (a) |  | 0.3(10) | -(-) | -(-) | -(-) | -(-) | -(-) |
|  | Hymenoptera | adult wasp |  | 0.8(20) | -(-) | -(-) | -(-) | -(-) | -(-) |
|  |  | Formicidae | Pseudomyrmecinae | 0.3(10) | -(-) | -(-) | -(-) | -(-) | -(-) |
|  | Arachnida | Acarina | Ixoidae | 1.5(30) | -(-) | -(-) | -(-) | -(-) | -(-) |
